# Risk of Tics with Psychostimulants for ADHD

**DOI:** 10.15844/pedneurbriefs-29-12-6

**Published:** 2015-12

**Authors:** J. Gordon Millichap

**Affiliations:** 1 Division of Neurology, Ann & Robert H. Lurie Children's Hospital of Chicago, Chicago, IL; 2 Departments of Pediatrics and Neurology, Northwestern University Feinberg School of Medicine, Chicago, IL

**Keywords:** Psychostimulant Medications, Methylphenidate, Amphetamine, Tics, ADHD, Meta-Analysis

## Abstract

Investigators at Yale University, New Haven, CT, conducted a meta-analysis to examine the risk of new onset or worsening of tics caused by psychostimulants used in the treatment of children with ADHD.

Investigators at Yale University, New Haven, CT, conducted a meta-analysis to examine the risk of new onset or worsening of tics caused by psychostimulants used in the treatment of children with ADHD. A PubMed search identified 22 double-blind, randomized, placebo-controlled studies involving 2,385 children with ADHD. New onset tics or worsening of tic symptoms were reported in 5.7% of the psychostimulant group and in 6.5% of the placebo group. The risk associated with psychostimulant treatment was similar to that observed with placebo (p=.962). The risk was not affected by type of psychostimulant, short or long-acting, dose, duration of treatment, nor by participant age. Crossover studies were associated with a significantly greater risk of tics with psychostimulant use compared to parallel group trials. Meta-analysis of controlled trials does not support an association between new onset or worsening of tics and psychostimulant use. The authors conclude that tics occurring during treatment of ADHD are more likely to be coincidental rather than caused by psychostimulants. The meta-anatysis findings provide strong support for rechallenging children who develop tics that are temporally related to the initiation of psychostimulants. [1]

COMMENTARY. Neurologists began to recognize tics as an organic disorder in the 1970s, shortly after the introduction of methylphenidate for the treatment of ADHD [2]. US FDA labeling warned against the use of stimulants in children with a personal or family history of tics. Since1983, psychostimulants are required to list tics or a family history of tics as a contraindication to their use for treatment of ADHD. Since then, many controlled trials have demonstrated no effect of psychostimulants on tics, and psychostimulants are as effective in treating ADHD and comorbid tics as in children with ADHD alone [3]. Symptoms of ADHD typically cause greater impairment in academic performance, social relationships, and neuropsychological performance, especially executive functioning, when ADHD is present in children with tics, an argument in favor of the use of psychostimulants.

Notwithstanding the clear evidence from meta-analysis that psychostimulants are innocent as a cause of tics in children with ADHD, the experience of the treating neurologist will often favor contrary conclusions and the avoidance of stimulants in a child who develops tics de novo or has significant worsening of tics when starting stimulants. In an editorial, recommendations for the management of this dilemma are outlined: 1) avoid use of stimulants when other causes of inattention (eg anxiety) are present; 2) explain the rates of tics as an adverse event (20% in children with pre-existing tics and 6% in those who have ADHD without tics); 3) tic occurrence waxes and wanes and may even complicate the introduction of a medication such as clonidine, sometimes used to treat tics [4].

## References

[1] Cohen SC, Mulqueen JM, Ferracioli-Oda E, Stuckelman ZD, Coughlin CG, Leckman JF (2015). Meta-Analysis: risk of tics associated with psychostimulant use in randomized, placebo-controlled trials. J Am Acad Child Adolesc Psychiatry.

[2] Denckla MB, Bemporad JR, MacKay MC (1976). Tics following methylphenidate administration. A report of 20 cases. JAMA.

[3] Bloch MH, Panza KE, Landeros-Weisenberger A, Leckman JF (2009). Meta-analysis: treatment of attention-deficit/hyperactivity disorder in children with comorbid tic disorders. J Am Acad Child Adolesc Psychiatry.

[4] Friedland S, Walkup JT (2015). Meta-assurance: No tic exacerbation caused by stimulants. J Am Acad Child Adolesc Psychiatry.

